# Primary Small-Cell Carcinoma of the Palate with Cushing's Syndrome: A Case Report

**DOI:** 10.1155/2012/539306

**Published:** 2012-10-21

**Authors:** Yingqiu Song, Zhenyu Lin, Lingjuan Chen, Gang Wu

**Affiliations:** Cancer Center, Union Hospital, Tongji Medical College, Huazhong University of Science and Technology, Wuhan 430000, Hubei, China

## Abstract

We report a 24-year-old woman presenting with a relapsed soy-bean-size tender mass at the junction of the soft and hard palate and a history of palatine tumor of small cell carcinoma. Reexcision surgery was performed and histopathological features were consistent. The patient was treated with six cycles of chemotherapy consisting of etoposide and cisplatin. After one year, the patient developed bone metastases and Cushing's syndrome, and successfully recovered with subsequent chemotherapy with irinotecan and cisplatin plus radiotherapy. There was no evidence of recurrence or metastasis for more than three years. Small cell carcinoma originating in the head and neck region has been reported to be highly aggressive and has a poor prognosis. This is the first case report of a patient with relapsed primary small cell carcinoma of the palate and successfully treated with second-line chemotherapy and local radiotherapy.

## 1. Introduction

Extrapulmonary small-cell carcinoma (EPSCC) is a relatively rare disease accounting for 0.1–0.4% of all malignancy and 2.5–4% of small-cell carcinoma [1, 2]. In the head and neck regions, the most common primary subsites are the salivary gland, nasal cavity, paranasal sinus, tonsil, larynx, and tongue [3, 4]. However, to our knowledge, small-cell carcinoma has never been reported to occur in the palate before. We presented a case of relapsed palate small-cell carcinoma accompanied with Cushing's syndrome and successfully treated with chemoradiotherapy.

## 2. Case Presentation

A 24-year-old woman had a history that included the excision of palate tumor in an outside hospital in February 2008. The tumor was a 2.5 × 1.5 × 1.5 cm mass, and the histopathologic examination showed small, round neoplastic cells with scant cytoplasm and hyperchromatic nuclei. The diagnosis of small-cell neuroendocrine carcinoma of the palate was determined and no adjuvant chemotherapy or radiotherapy was performed after surgery. The patient was free from tumor until November 2008, when signs of recurrence in the primary lesion were detected during a follow-up visit. Then she was transferred to our hospital for further treatment.

Physical examination found a soy-bean-size, tender mass at the junction of soft and hard palate fixed to the underlying tissues. No clinical evidence of lymphadenopathy was present. 

**Figure 1:**
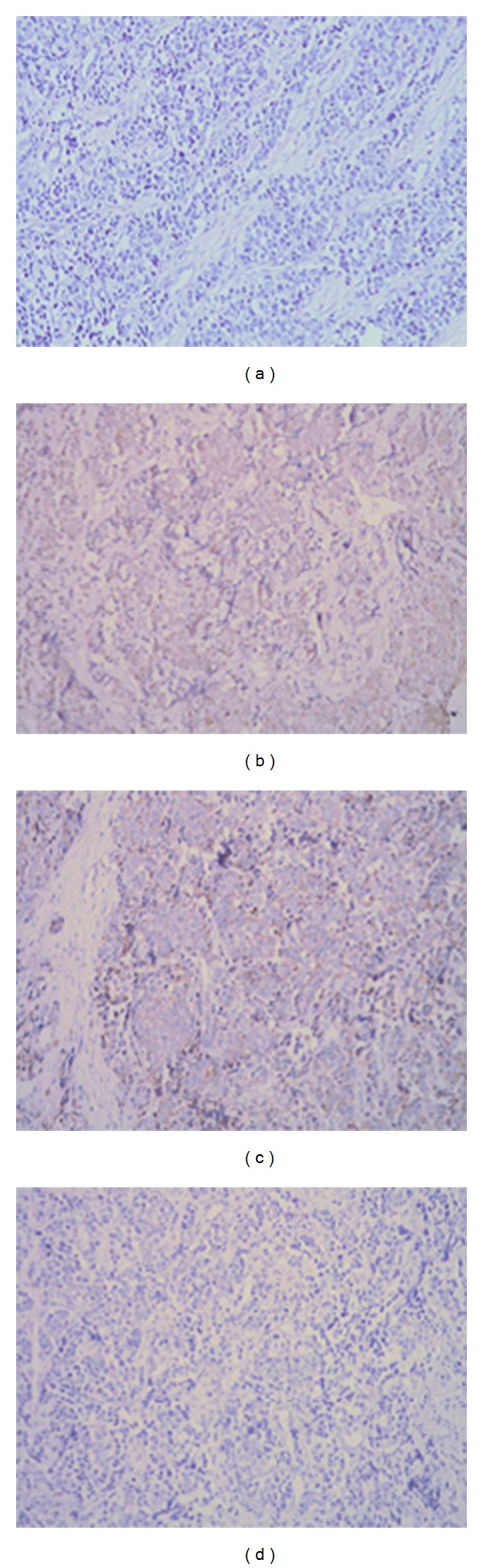
Photomicrographs showing histological and immunohistochemical findings. (a) The tumour is composed of small-cells with round to fusiform shape, scanty cytoplasm with fine granular nuclear chromatin, absence of nucleoli, and with high mitotic activity (H & E; ×200). The tumour cells were immunohistochemically positive for (b) cluster of differentiation 56 glycoprotein, (c) synaptophysin (a neuroendocrine marker) (×200), and (d) CgA (×200).

Magnetic resonance imaging (MRI) revealed an ill-defined mass of the palate. Computer tomography (CT) scans of the brain, chest, and abdomen, as well as radionuclide bone scan (ECT) were carefully examined to look for signs of metastatic disease. There were no evident metastases at regional lymph nodes or any distant organs. Again she underwent extensive resection of palatine tumor on February 14, 2008. The postoperative pathology reports were compatible with the previous diagnosis of small-cell carcinoma. Immunohistochemical staining was positive for cluster of differentiation 56 glycoprotein (CD56), synaptophysin (Syn), and chromogranin A (CgA) (Figure 1); CD34, CD31, S-100, VIM, GFAP, DES, and MyoD were negative. Postoperatively, the patient received six cycles of adjuvant chemotherapy with etoposide and cisplatin. Upon completion of the chemotherapy, all oncological investigations, including abdominal, pelvic, and chest CT, revealed no evidence of recurrence. Outpatient follow up of laboratory and radiographic studies was performed at regular intervals.

The patient demonstrated a favorable prognosis. However, after one year, two nontender, firm, well-circumscribed nodules measuring 2.5 × 2 cm and 2 × 3 cm were noted on the left clavicular head and manubrium. Thus she was readmitted to our hospital. The initial laboratory data on admission were normal. ECT showed multiple-bone metastases including the third lumber vertebral body and the left clavicle. Irradiation of the two involved lesions were given from November 23, 2009 to December 18, 2009, 30 Gy in 10 fractions to the lumbar vertebrae and 50 Gy in 25 fractions to her left clavicular and sternum. Subsequently, she received six cycles of treatment with irinotecan and cisplatin.

**Figure 2:**
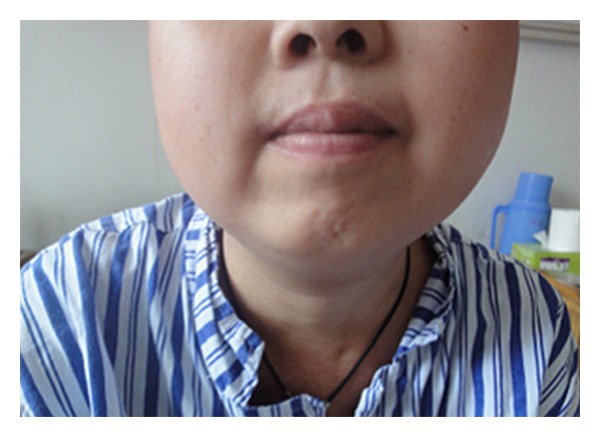
Clinical photograph of the “moon face”.

Meanwhile, during the radiotherapy the patient also developed symptoms of weakness, hand numbness, finger twitching, facial swelling, and nocturia (Figure 2). Her laboratory evaluations showed refractory hypokalemia with a random potassium level of 2.1 to 3.3 mmol/L (reference range: 3.5 to 5.5 mmol/L), hypercalcemia with a free calcium level of 2.89 to 2.98 mmol/L (reference range: 2.18 to 2.9 mmol/L) and hyperglycemia with a fasting blood-glucose of 8.5 mmol/L (reference range: 3.9 to 6.0 mmol/L). A random serum cortisol level showed significant elevation (>400 *μ*g/L, reference range 37–194 *μ*g/L), and the urinary 17-hydroxycorticoids (17-OHCS) was also increased (173 *μ*g/L, reference range 35–55 *μ*mol/24 h). An overnight, low-dose dexamethasone suppression test revealed a failure to suppress cortisol. Thus a diagnosis of Cushing's syndrome caused by the ectopic production of ACTH was made. She underwent symptomatic treatment during the radiotherapy and the Cushing's syndrome was controlled well. The patient is currently well and there has been no evidence of metastasis for three years.

## 3. Discussion

Extrapulmonary small-cell carcinoma (EPSCC) has been increasingly recognized as a distinct clinical pathologic entity since its initial description in 1930 by Duguid and Kennedy [5]. Over the last decades, the number of reported cases in all sites of the body has increased several folds. However, very few cases of the head and neck are described in the literature, and primary small-cell carcinoma of the palate is extremely uncommon. EPSCC shares the similarity in its histology with that of the small-cell lung carcinoma, which is characterized as round or spindle-shaped small-cells with hyperchromatic nuclei, inconspicuous nucleoli, and sparse cytoplasm. Some EPSCCs are classified as part of the neuroendocrine family of tumors with neurosecretory granules. A variety of immunohistochemical markers, including cytokeratin, neuron-specific enolase (NSE), epithelial membrane antigen (EMA), carcinoembryonic antigen (CEA), chromogranin A, and synaptophysin, are helpful for the diagnosis of EPSCC [6–8]. Our case was confirmed to be a neuroendocrine carcinoma of the palate by the pathological and immunohistological examinations.

The prognosis of EPSCC is poor with a 5-year survival of 13% and a median survival from diagnosis of 14.5 months, which has also been shown to be partially dependent on the primary disease site [9, 10]. In EPSCC of the head and neck, tracheal small-cell carcinoma shows the worst prognosis, laryngeal and hypopharyngeal small-cell carcinomas have intermediate survival, while those of the nose, paranasal sinuses, and parotid gland have the best prognosis. The lungs, liver, and bone are the common sites for distant metastases from head and neck small-cell carcinomas [11].

The optimal therapeutic strategy of EPSCC is still unknown, mainly due to the relative rarity of the tumor. However, since micrometastases are usually present at the time of diagnosis, surgery alone is associated with a high rate of recurrence. For the majority of patients with first diagnosed EPSCC, radical surgery or definite radiotherapy has been frequently employed. Because of its aggressive malignancy with a high tendency of local recurrence and metastatic spread, multimodality therapy has become increasingly applied, including chemotherapy, radiotherapy, and possibly surgery depending on the extent of disease or primary site. The chemotherapeutic regimens of EPSCC are similar to those utilized in SCLC, and the combination of etoposide and cisplatin (EP) is the first line treatment with a response rate of 69% [12]. Radiotherapy also has an effective palliative role in many sites. In our case, due to the lack of knowledge of EPSCC, no chemotherapy or radiotherapy was complemented with surgery after the first treatment. Actually, the clinical absence of cervical and distant involvement does not exclude the presence of micrometastases, which may become clinically evident after a few months. Consequently, the patient relapsed at the primary site after 8 months. Considering the dimension and site of the mass, reoperation and adjuvant chemotherapy were performed.

Ectopic hormone production is observed in 10–15% of small-cell carcinoma, and occurs less frequently in small-cell carcinoma of the head and neck than in small-cell lung carcinoma [13, 14]. In our case, as the disease progressed, the patient suffered from severe ectopic ACTH syndrome (Cushing's syndromes). Clinically, the overt ectopic ACTH syndrome may be mainly due to rapidly progressive small-cell carcinoma, and usually manifests as a rapid onset of symptoms including profound pigmentation, weakness, hypertension, hypokalemia, hyperglycemia, polyuria, while there is often little obesity and absence of classically Cushingoid appearance. In contrast, the occult ectopic ACTH syndrome usually due to more indolent tumours, such as bronchial, thymic, and pancreatic carcinoids, shows typical Cushingoid appearance like moon face, central obesity, and buffalo hump [15]. The optical treatment of ectopic ACTH syndrome is generally considered to eliminate all of the cancerous tissue that is secreting ACTH. The choice of cancer treatment-surgery, radiotherapy, chemotherapy, immunotherapy, or a combination of these treatments depends on the type of cancer and how far it has spread [14]. Obviously overt ectopic ACTH syndrome occured in our patient, and the Cushing's syndrome was alleviated after the radiotherapy and chemotherapy combined with supportive measures like correction of electrolyte imbalance. The patient is currently well without any evidence of recurrence for more than three years. 

## 4. Conclusion

Primary palatine small-cell carcinoma is extremely rare. After complete resection of the tumor, adjuvant chemotherapy should be provided due to the high incidence of occult metastases. The ideal treatment of small-cell carcinoma of the palate should be the combination of surgery, radiotherapy, chemotherapy, or supportive care depending on the stage of the disease.
